# The role of neutrophil extracellular traps in necrotizing enterocolitis

**DOI:** 10.3389/fped.2023.1121193

**Published:** 2023-03-15

**Authors:** Michaela Klinke, Hala Chaaban, Michael Boettcher

**Affiliations:** ^1^ Department of Pediatric Surgery, University Medical Center Mannheim, University of Heidelberg, Mannheim, Germany; ^2^ Department of Pediatrics, The University of Oklahoma Health Sciences Center, Oklahoma, OK, United States

**Keywords:** neutrophils, NETs (neutrophil extracellular traps), NEC (necrotizing enterocolitis), intestinal disease

## Abstract

Necrotizing enterocolitis (NEC) continues to be one of the most common causes of mortality and morbidity in preterm infants. Although not fully elucidated, studies suggest that prematurity, formula feeding, imbalanced vascular supply, and altered bacterial colonization play major roles in the pathogenesis of NEC. NEC is characterized by increased cytokine release and leukocyte infiltration. Recent data from preterm infants and animal models of NEC suggest that neutrophil extracellular traps (NETs) are released in intestinal tissue. The contribution of NETs in the pathogenesis and/or prevention/treatment of this disease continues to be controversial. Here, we review the available data on NETs release in NEC in human patients and in different NEC models, highlighting their potential contribution to pathology and resolution of inflammation. Here, we review the available data on NETs release in NEC in human patients and the different NEC models, highlighting their potential contribution to pathology or resolution of inflammation.

## Introduction

Necrotizing enterocolitis (NEC) is one of the most devastating diseases in the neonatal intensive care unit. This inflammatory bowel disease primarily affects preterm infants, with an incidence of 7%–12% in neonates born less than 1,500 g (1). Importantly, the incidence is steadily increasing, as improvements in neonatal care lead to enhanced survival of premature infants (2–4.) NEC is associated with mortality rates of up to 30% in very low birth weight infants (5), and up to 80% in the most severe cases (fulminant NEC) (6). Moreover, survivors of NEC are at increased risk of long-term morbidities such as growth failure, short bowel syndrome, and neurodevelopmental delay, all of which increase the physical and psychological burdens for patients and their families (7, 8).

Despite decades of investigations, the pathogenesis of NEC remains inconclusive, perhaps since NEC may not be a single disease, but comprised of several entities (e.g., classic NEC, ischemic intestinal necrosis, food protein intolerance enterocolitis syndrome) (9–11) NEC pathogenesis is multifactorial with prematurity, formula feeding, and dysregulation of perfusion, as well as dysbiosis, playing major roles. In preterm neonates, developmental immaturity of the mucosal barrier and increased expression of toll-like receptor (TLR) 4 in the intestinal epithelium render the gut highly reactive to stimuli (12, 13). The net effect is exaggerated inflammatory cytokine/chemokine release, leukocyte infiltration, epithelial necrosis, altered epithelial barrier, and bacterial translocation across the lumen (14). Of particular interest, TLR4 expression can be reduced by breast milk feeding (15). This excessive TLR4 expression in response to the dysbiotic microbiome can lead to the death of intestinal epithelial cells through apoptosis and necroptosis, as well as impaired mucosal restitution, which in severe cases leads to intestinal perforation, multi-organ failure, and potentially death (16).

Notably, NEC is not solely a disease of the abdomen, rather it is a multisystemic disease that can also affect other organ systems (17–19). Systemic reviews have demonstrated that NEC is an independent risk factor for neurocognitive developmental delay and poor neurocognitive outcomes (20, 21). Moreover, studies suggest that common morbidities of the preterm infant such as bronchopulmonary dysplasia (BPD) and brain damage are affected by the development of NEC necrotizing enterocolitis, through interactions known as the “Gut-Lung-Axis” and “Gut-Brain-Axis”, respectively (18, 19). Recently, it has been reported that the excessive immune response *via* TLR4 and neutrophil activation are associated with increased damage to not just the intestine but also the lung and brain tissue, suggesting a potential role of neutrophils in distant organ injury in NEC (17–19). Suggesting a potential role of neutrophils in distant organ injury in NEC.

## Neutrophils in necrotizing enterocolitis

Neutrophils are the most abundant immune cells and first-line responders of the innate immune system (22, 23). As polymorphonuclear cells, neutrophils are very motile, which enables them to migrate from peripheral vasculature into the tissue of recruitment (23, 24). One of the key functions in NEC pathogenesis is the activation of intestinal epithelial cell toll-like receptor 4 (TLR4) which leads to accelerated apoptosis of enterocytes and reduced rate of healing through impaired intestinal restoration and proliferation. Upregulation in TLR4 expression results in the production of pro-inflammatory cytokines and chemokines leading to the recruitment of neutrophils to the location of inflammation (25). Neutrophil infiltration has been long recognized in NEC tissues. However, the beneficial and detrimental contributions of these cells specifically in this disease remain unclear (11, 26, 27). Neutrophils seem to be critical for mucosal homeostasis as NEC is aggravated by neutrophil depletion in a murine model of NEC (28). However, excessive recruitment and activation of neutrophils could also promote injury and exacerbate disease including mutual upregulation of TLR4 and neutrophil activation (29).

**Figure 1:**
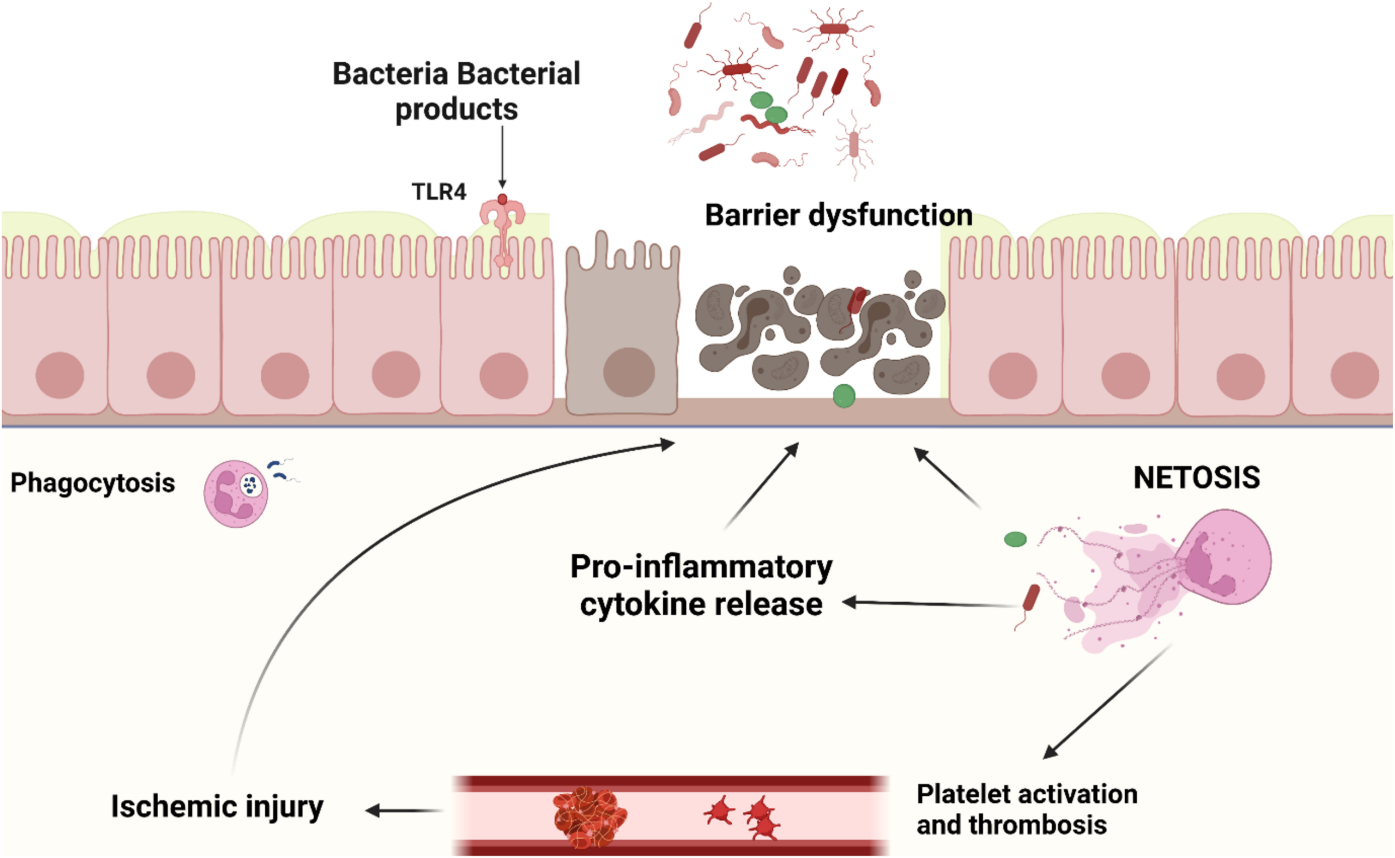
Neutrophil activation and NETs release in intestinal tissue of NEC patients occur as a result of various stimuli such as pathological bacteria, PAMPs and ROS. Though NETs and their components are important to prevent bacterial translocation through the intestine, dysregulated NETs release leads to adjacent epithelial and endothelial injury, cytokine release, platelet activation and thrombus formation. This would result in a vicious cycle of tissue necrosis, further immune activation, ischemia reperfusion injury, uncontrolled inflammation, multi-organ failure, and death.

Upon contact with pathogens, neutrophils can react *via* (1) phagocytosis, (2) production of oxidative bursts like reactive oxygen species (ROS), and (3) degranulation and/or (4) formation of neutrophil extracellular traps (NETs) (30). NETs are large extracellular networks consisting of DNA fibers and spherical proteins. The protein contents of NETs include histones, neutrophil elastase (NE), myeloperoxidase (MPO), defensin, calprotectin, cathepsin G, protease 3, and actin, lactoferrin, gelatinase, lysozyme C, and cathelicidins (24, 31–33). Neutrophils release NETs *via* multiple mechanisms: (1) NETosis, a programmed cell death pathway distinct from apoptosis, pyroptosis, necroptosis, or ferroptosis, (2) non-lytic discharge of parts or their entire nucleus, and (3) mitochondrial DNA release, providing an additional DNA source for NET formation (34). The main processes involved in NETosis are neutrophil activation, cytoplasmic granule dissolution, neutrophil protease activation, chromatin decondensation, and swelling, followed by plasma membrane rupture. NETs are released after histones are citrullinated by peptidyl arginine deiminase 4 (PAD4) (35). The function of those structures is to capture pathogenic microorganisms and enhance phagocytosis by macrophages thus preventing the spreading of infection (24, 36–38). NETs are normally cleared by plasma DNAse 1 followed by removal by macrophages. Inappropriate or delayed clearance of NETs or NET components specifically the associated histones and proteases contribute to pathological conditions like sepsis (39), thrombosis (40), transfusion-associated acute lung injury (41), cancer development and metastasis (42), autoimmune diseases (23, 43), and impaired wound healing (44) - mostly through induction of INF, proinflammatory cytokines, and the NLRP3 inflammasome (45). A major mechanism for the cytotoxic properties of histones in NETs is through direct binding to the plasma membrane causing calcium influx and loss of membrane barrier function (46–48). Histones also activate TLRs 2, 4, 9 leading to cytokine production, leukocyte recruitment, and tissue injury (49–51). Furthermore, extracellular histones in NETs stimulate platelet adhesion and coagulation (52), which in severe cases can lead to multi-organ failure due to micro thrombosis, decrease microvascular perfusion, and subsequent tissue damage (53). This has been demonstrated by the association of NETs with various thrombo-inflammatory diseases such as stroke, autoimmune diseases, sepsis, lung injury (i.e., COVID-19), diabetes, and ischemia-reperfusion injury of the intestine and testicles (54–60). To further illustrate their double-edged nature, NETs released in the gut have been shown to reduce the translocation of bacteria and support the healing of the intestinal mucosa. On the other hand, excessive NETs formation can damage the barrier function of the intestinal mucosa and thus play a key role in the development of a variety of intestinal diseases (23, 42) (Figure 1).

In neonates, neutrophils exhibit an intrinsic delay in NET formation but are capable of releasing functionally competent NETs (61–64). In a series of elegant experiments, Yost et al. showed that neutrophils isolated from term and preterm infants fail to form NETs in response to ROS, LPS, and bacteria after an hour of incubation. This defect in NET formation was associated with a reduction in extracellular bacterial killing *in vitro* compared to neutrophils isolated from adults (62). Such differences could explain the increased susceptibility of neonates and preterms in particular to sepsis and infection. To explore the mechanism for this blunted neonatal NET deployment, Yost et al. identified peptides in cord blood from preterm and term infants that inhibit NETosis (63) *in vitro* and *in vivo* and appear to be an endogenous regulator of NET generation. Importantly, the authors assessed the ability of neutrophils from preterm neonates longitudinally over the first 28 days after birth for NET formation in response to LPS. NET formation was not demonstrated until day 3 after birth and reached maximum capacity between days 3 and 14. Proteomic analysis identified neonatal NET-inhibitory factor (nNIF) detected in plasma from cord blood of term and preterm infants in the first days after birth and is absent in plasma of adults. Importantly, nNIF use in animal models of inflammation and polymicrobial sepsis improved survival and multiorgan injury, supporting existing evidence that NETs are effectors of collateral vascular and tissue injury in certain pathologies.

## Necrotizing enterocolitis and neutrophil extracellular traps

Previous studies showed that NETs release occurs in tissues, serum, and stool of infants and animal models of NEC (26, 65–67). In a prospective pilot study, McQueen et al. showed that infants diagnosed with NEC had increased fecal calprotectin levels compared to infants with NEC “ruled out”. Further analysis using immunohistochemistry, showed an association between calprotectin staining, neutrophil activation markers, and NETs staining in the intestinal tissues of infants with surgical NEC. These data suggest that fecal calprotectin is released, at least in part, as a result of neutrophil infiltration, activation, and potentially NET formation in the intestinal tissue of infants with NEC (67). Other studies later confirmed NETs release in NEC patients and animal models of NEC. Nguyen et showed that preterm infants with NEC and sepsis had higher levels of cell-free DNA (cfDNA), a surrogate marker of circulating NETs, compared to controls (66). Similarly, Chaaban et al. showed increased levels of nucleosomes (histones-DNA), also a surrogate of NETs release, in the serum of infants with NEC stage II and above compared to gestational age-matched controls (65). Analysis of intestinal tissue confirmed neutrophil activation and NET release by immunohistochemical staining of intestinal tissue from preterm infants and a mouse model of NEC. Vincent et al. demonstrated that the pathogenesis of NEC is likely a NET-dependent process (26). They showed that markers of neutrophil activation and NET formation in both serum and histology directly correlate with NEC manifestation, severity, and mortality in a murine model of NEC that utilizes intermittent hypoxia/LSP, and formula feeding. Furthermore, the prevention of NET formation by PAD4 inhibition, using Cl-amidine, significantly reduced NEC histological injury, inflammation, and mortality in the model. The same group further showed that degradation of extracellular DNA in NETs by systemic application of DNase1 leads to a significant reduction in NEC severity, and mortality, suggesting an important role in the pathogenesis of NEC (68). The crucial role of NETs in NEC pathogenesis is further emphasized by the results of Klinke et al. wherein neutrophil concentrations of mice were elevated to match those of human neonates as a method to optimize intestinal injury in the NEC model. Of particular interest is that the NEC severity, tissue damage, and inflammation were significantly reduced, and similar to mice in the control group, in ELANE gene knockout pups, who are incapable of forming NETs (ELANE gene encodes for neutrophil elastase, so knockout results in lack of a key enzyme in NET formation) (69).

These data are in line with the recent studies that suggest, that the degradation of NETs by DNase1 significantly reduces gut-related inflammation, apoptosis of intestinal epithelial cells, and intestinal damage (23). Martinod et al. also showed that suppression of NETs formation by PAD4 inhibition does not impair the ability of neutrophil granulocytes to defend against pathogens and, in particular, does not lead to higher bacteremia or mortality rates in a model of polymicrobial sepsis (70). Moreover, Silva et al. showed that another method of inhibition of NET formation by disulfiram improves organ function and lethality in sepsis (71). Finally, the neonatal NET-inhibitory factor (nNIF) appears to inhibit NET formation in fetuses and neonates in the first days after birth (63). Whether the maturation of NET formation which coincides with the timing for the development of NEC, plays a role in the pathogenesis of NEC, is yet to be determined. It is possible that preterm neonates develop NEC after a period of time, when the protective effects of the nNIF wear off.

In contrast, the use of PAD4 inhibition in another model of NEC characterized by bacteremia, known as the dithizone/klebsiella NEC model was associated with worsened outcomes. NETs inhibition in this model using cl-amidine was associated with increased inflammatory response, increased bacterial translocation, and mortality in the NEC mice (67). Similarly, Saha et al. showed that PAD4-dependent NET generation is indispensable for intestinal clearance of *Citrobacter rodentium* enteric infection, highlighting the beneficial effects of NETs release in an infectious context (72). These contradicting results strongly suggest that the effects of NET formation may be disease- and model-specific, and in NEC, they depend largely upon the level of intestinal bacterial translocation. They appear to play an integral role in innate defense, especially early on in the clearance of bacterial and bacterial products.

## Conclusion

Our current understanding suggests that NETs may be a double-edged sword. They are relevant in the immune defense against pathogenic agents. However, excessive NET ormation induces hyperinflammation, tissue damage, and thrombo-inflammation, contributing to the pathogenesis of a wide variety of diseases such as sepsis, NEC, ARDS, lung injury in COVID-19, ischemia-reperfusion injury, and various oncological diseases. The effect of NETs in pathological conditions is perhaps disease and model specific. In NEC, it is likely dependent on the level of intestinal bacterial translocation. NETs seem crucial in the early phase of the disease to battle bacteremia and reduce bacterial translocation in NEC. However, after the initiation of antibiotic therapy, it may be reasonable to try reducing NET formation through the use of agents like DNases to avert the hyperinflammatory damage caused by NETs. Future studies are needed to further investigate the role of NETs in NEC and other human diseases and explore how best to optimize the beneficial effects and minimize the detrimental effects of NETs for therapy in various human diseases including NEC.
